# Embodied Energy Use in China’s Transportation Sector: A Multi-Regional Input–Output Analysis

**DOI:** 10.3390/ijerph18157873

**Published:** 2021-07-25

**Authors:** Jing Li, Hong Fang, Siran Fang, Zhiming Zhang, Pengyuan Zhang

**Affiliations:** 1 College of Economics and Management, Taiyuan University of Technology, Taiyuan 030024, China; Jinglibuaa@yeah.net; 2 School of Economics and Management, Beihang University, Beijing 100191, China; fanghong@buaa.edu.cn (H.F.); cgfwzx@126.com (Z.Z.); aa1134151203@126.com (P.Z.); 3 School of Economics and Management, University of Chinese Academy of Sciences, Beijing 100190, China

**Keywords:** transport sector, embodied energy consumption, MRIO model, interprovincial energy transfer

## Abstract

Energy transition in the transport sector (TS) is important for the goals of achieving carbon peak by 2030 and carbon neutrality by 2060 in China. A number of scholars have conducted studies to identify the potential energy savings of the TS and to improve its energy efficiency. Most of them concentrate on the direct energy use (EU). The indirect EU along the supply chain of the TS is often neglected. In this paper, the direct and indirect energy use, i.e., the embodied EU, of China’s TS is measured by applying a multi-regional input–output (MRIO) model, where they are investigated from both the provincial and sectoral perspectives. Results show that intermediate use is the major driving force for the TS’s embodied EU in China. From the sectoral level, supply from sectors such as petroleum refining and coking and demand from the service sector are the main reasons of the TS’s embodied EU. From the provincial perspective, the TS’s embodied EU is driven by low transportation technologies in most provinces located in central and western China. By contrast, abundant economic and social activities are the primary reason for the TS’s embodied EU in most provinces in eastern China. In the terms of interprovincial transfer, the energy embodied in the TS flows from resource-intensive provinces located in central and western China to resource-deficient provinces in eastern China. In addition, a close geographical connection exists in the transfer. Finally, several strategies from the sectoral and provincial levels are provided for policymakers.

## 1. Introduction

**Figure 1:**
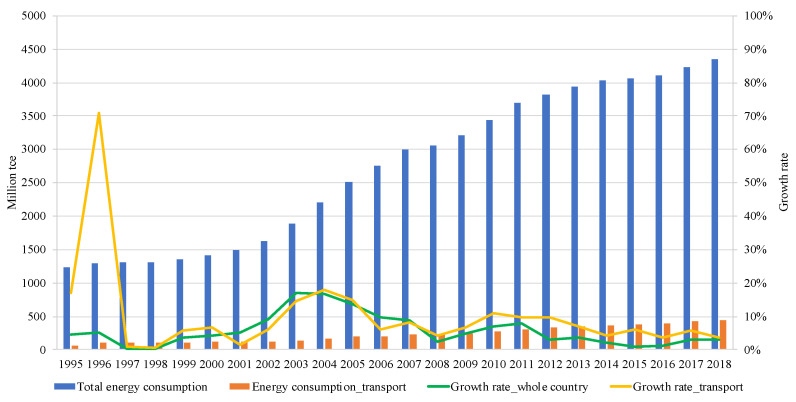
Energy consumption in China. Source: calculated based on China Energy Statistical Yearbooks.

In the current Chinese economy, a number of hot issues need to be dealt with, such as key technologies, international trade, urban and rural planning, and energy. Energy consumption (EC) has obtained great attention from the government. According to Figure 1, the EC in China has experienced an upward trend during 1995–2018, which increased from 1234.7 million tons of standard coal equivalent (Mtce) in 1995 to 4356.5 Mtce in 2018, an increase of nearly 3.5 times. Especially from 2002 to 2007, EC grew fast, its growth rate is more than 9% because of the rapid economic and urbanization development, as well as the rising population and resident income in China [1]. A negative impact of the increased EC is that it can lead to a large amount of greenhouse gas (GHG) emissions. To reduce the GHG emissions, China has made several mitigation commitments. Recently, President Xi stated at the general debate of the UN General Assembly that China would strive to reach carbon peak by 2030 and carbon neutrality by 2060.

To achieve the goals, Chinese governments have taken a series of measures aimed at sectors’ EC. TS is one of the high-energy-consuming sectors in China. Its EC has grown from 58.6 Mtce in 1995 to 436.2 Mtce in 2018 (see Figure 1). Due to the continuous improvement of the living standards of Chinese residents, this trend will continue [2]. Meanwhile, the share of its EC in the Chinese total has increased from 7.6% in 2009 to 10.0% in 2018 [3]. The rapidly increasing EC of the TS causes serious resource consumption and environmental pollution [4,5] and threats to public health [6]. To mitigate the negative influences, Chinese central and provincial governments have implemented a number of policies, such as development of urban public transport, energy consumption tax, automobile emission requirements, measures to restrict vehicles on the road, supporting consumers to buy small-displacement cars, and promoting new energy vehicles. [7,8]. Although the growth rate of the TS’s EC has declined from 10.8% in 2010 to 3.5% in 2018, its volume is still increasing [9]. Moreover, the growth of the TS’s EC is faster than the total in China when comparing the growth rate. The high EC issues rooted in China’s TS are still not completely resolved [10]. Therefore, it is necessary to investigate the EU of the TS.

Due to the energy-intensive characteristics, research on analyzing TS’s EC in China has been conducted by a number of scholars and primarily concentrates on three topics: EU evaluation and predication, measurement of energy efficiency, and analysis of factors influencing EU.

As for the first topic, for instance, Yin et al. [11] explore the future development of the TS in China from 2010 to 2095 and suggest that its EC and CO_2_ emissions would continue to grow if there were no policy to intervene. Through setting up a Transportation Mode–Technology–Energy–CO_2_ model, Wang et al. [12] investigate the relative influences of some implemented transport policies on EC and carbon dioxide emissions up to 2050 in China. Liu et al. [2] estimate the TS’s EU and air emissions during 2010–2050 with the help of a Long-range Energy Alternatives Planning system (LEAP) model. Ren et al. [13] conduct life cycle analysis of primary EC of hydrogen supply chains for fuel-cell vehicles in China. Yaqoob et al. [14] carry out a comprehensive comparison for different types of transport fuels for Pakistan, through undertaking various factors, such as fuel properties, production, consumption, emission of gases, engine performance, and economy.

As for the second topic, data envelopment analysis (DEA) is widely used to measure the energy efficiency of the TS. For example, Zhou et al. [15] apply an environmental DEA method to investigate energy efficiency and also quantify energy-saving potentials in China’s TS. Liu et al. [16] calculate the energy-environment efficiency of the TS in 30 Chinese regions through integrating non-radial DEA and window analysis. By using a parallel slack-based measure (SBM) DEA model, Liu et al. [17] evaluate the environmental efficiency of the land transport sector. Feng and Wang [18] introduce a global meta-frontier method to measure the energy efficiency and savings of Chinese TS. A new equilibrium efficient frontier DEA is utilized by Zhu et al. [19] to evaluate the energy and environmental efficiency of China’s transportation sectors. Wei et al. [20] combine stochastic multicriteria acceptability analysis (SMAA-2) with DEA to analyze the energy and environmental efficiency of Chinese transportation sectors in the presence of uncertain CO_2_ emission data.

For the third topic, logarithmic mean Divisia index (LMDI) is a frequently employed approach to investigate the factors affecting TS’s EU. For instance, Chung et al. [1] decompose EU changes from 2003 to 2009 into four factors, i.e., the effects of activity, structure, intensity, and energy mix. Mraihi et al. [21] determine the road transportation’s driving factors for the EC changes. Achour and Belloumi [22] identify the driving factors of energy use of transportation in Tunisia during 1985–2014. Isik et al. [23] evaluate and reveal the influential factors on CO_2_ emissions in the Turkey’s transportation sector between 2000 and 2017. In addition, other methods are also used to study the transport-related energy and environment issues. Chen and Lei [24] apply the path analysis model to evaluate the direct and indirect effects of factors on carbon emissions of the transport sector in Beijing. Li et al. [25] examine the influence of environmental regulations on transportation and also compute its green total factor productivity during 2000–2010. Utilizing annual data from 2015 to 2012, Li et al. [26] explore the effect of tourism investments on the 32 OECD economies’ energy efficiency of transportation. Wang et al. [27] develop an integrated method combining the Tapio decoupling approach with the decomposition technique to investigate the decoupling state between economic output and carbon emission in China’s transport sector and to analyze its driving factors. A hybrid decomposition analysis framework is proposed by Xu et al. [28] to analyze the growth sources of EU in the passenger transport sector, which concludes that transport service increase and transport structure shift are the main reasons for the increase in EU, while energy efficiency improvement is a major cause to counteract the increase. Employing the extended STIRPAT (stochastic impacts by regression on population, affluence, and technology) and GTWR (geographically and temporally weighted regression) model, Liu et al. [29] investigate the influence of driving factors on the carbon emission intensity of China’s transportation sector.

Although the Chinese TS’s EC has been analyzed and predicted in many studies, they mainly focus on the direct EU during the process of vehicle running. The indirect EU induced by the demand from itself and its downstream sectors is often ignored. In addition, lack of understanding of the interprovincial transfer of EU would lead to biased or unfair policies and measures for the TS in different provinces. Thus, it is essential to explore the direct and indirect EC of the TS and its interprovincial energy transfer.

The multi-regional input–output (MRIO) model is an effective and appropriate approach, which can measure and analyze the direct and indirect energy consumption (i.e., embodied or total energy use) by considering the differences of regions and sectors at the same time [30]. Due to the practicality and effectiveness, it has been applied to deal with different issues at the level of world, nations, and regions. For example, Usubiaga and Acosta-Fernández [31] introduce the MRIO model to quantify the carbon emissions based on the territory and the residence principles. Using an environmental MRIO model, Wu and Liu [32] investigate China’s thirty provinces’ imbalance, unfairness, and pressure in the ecological footprints. Wang et al. [33] analyze the environmental damage transfers embodied in the Chinese provincial trade in 2007. Zhan et al. [34] employ this method and measure the ecological footprint transfer between the Beijing–Tianjin–Hebei region and the other areas in China. In addition, some scholars utilize it to analyze issues at the industrial level, such as the construction sector studied by Chang et al. [35,36], the coal industry by Li et al. [37], or the iron and steel industry by Peng et al. [38]. Hue and Tuyet [39] use IO tables to investigate the energy intensity of transport service sectors in Vietnam; however, the single-regional IO is applied in this research without considering interregional energy transfer in the TS. There are few studies utilizing the MRIO model to calculate the EC embodied in Chinese TS and to analyze its energy transfers at the level of regions and sectors.

Based on the above analysis, the objective of this study is to have a holistic investigation and analysis for the embodied EC of Chinese TS through applying the MRIO model. The major contributions are listed as follows: (1) A 2012 Chinese MRIO table and the MRIO model are introduced to quantify the volume of the TS’s embodied EU, where regional heterogeneities are taken into account. (2) The EU embodied in interprovincial trade and intersectoral flow is also calculated and explored, which would assist readers to have a deep insight of the TS’s energy use due to the trade among regions and the input–output relationship of sectors. (3) Strategies that fundamentally handle the high-energy intensive problems in the TS are proposed from the perspectives of sectoral and provincial level, which could provide references for policymakers to make reasonable policies.

The rest of this study is arranged as follows: the basics of the MRIO model are introduced, and the data sources are presented in Section 2. Then, Section 3 analyzes the calculation results of transport sector’s embodied energy use in 2012. Discussions and policy implications are provided in Section 4. Finally, the conclusions are summarized in Section 5.

## 2. Method and Data Processing

### 2.1. MRIO Model

In a MRIO model, input–output tables and trade flow tables, which represent financial transactions among sectors and the value of exports and imports within a country or the world, respectively, are integrated into a coherent accounting framework [40]. Strengths of this methodology have been assessed and summarized in some research works [41]. For example, it can track the influences of supply chains across multiple sectors in multiple countries or regions; total resource use can be divided based on detailed production and consumption activities. The MRIO model also has weaknesses, for instance, the number of sectors is relatively fixed, which compromised on the number of countries; time series data are limited due to the huge raw data collection and significant manual labor and time. Overall, MRIO is a useful approach that measures all indirect impacts induced by upstream supply or downstream demand.

In this section, a MRIO model is constructed to study the TS’s EC embodied in interprovincial trade and intersectoral flows in China. It is an effective methodology to quantify environmental influences from a top–down perspective [42,43]. To concentrate on the domestic interregional links, the imports item has been removed from the MRIO table [44]. Table 1 shows the revised format of the MRIO table. The basic monetary balance of the MRIO model is expressed as follows:

(1) $$x_{i}^{r}=\overset{m}{\underset{s=1}{\sum }}\overset{n}{\underset{j=1}{\sum }}z_{ij}^{rs}+\overset{m}{\underset{s=1}{\sum }}\overset{p}{\underset{q=1}{\sum }}d_{iq}^{rs}{+ex}_{i}^{r}{+o}_{i}^{r}$$

where $x_{i}^{r}$ stands for total output of the sector *i* in province *r* (*i* = 1, 2, …, *n*; *r* = 1, 2, …, *m*). $z_{ij}^{rs}$ represents the intermediate monetary input from sector *i* in province *r* to sector *j* in province *s*. $d_{iq}^{rs}$ represents the monetary value of the *q*th category of domestic final uses in province *s* provided by the sector *i* in province *r* (*q* = 1, 2, …, *p*). The domestic final uses have totally five categories (i.e., *p* = 5), i.e., rural household consumption, urban household consumption, government consumption, fixed capital formation, and inventory increase. ${ex}_{i}^{r}$ is the export of sector *i* in province *r*, which represents foreign demand for the products of sector *i*. $o_{i}^{r}$ stands for the monetary value of other terms of sector *i* in province *r*. $\overset{m}{\underset{s=1}{\sum }}\overset{p}{\underset{q=1}{\sum }}d_{iq}^{rs}$, ${ex}_{i}^{r}$, and $o_{i}^{r}$ are identified to represent the final demands of sector *i* in province *r*.

When energy flows are introduced into the MRIO table, an energy balance equation is built by [45]

(2) $$e_{i}^{r}x_{i}^{r}=\overset{m}{\underset{s=1}{\sum }}\overset{n}{\underset{j=1}{\sum }}e_{i}^{r}z_{ij}^{rs}+\overset{m}{\underset{s=1}{\sum }}\overset{p}{\underset{q=1}{\sum }}e_{i}^{r}d_{iq}^{rs}{+e}_{i}^{r}{ex}_{i}^{r}{+e}_{i}^{r}o_{i}^{r}=\overset{m}{\underset{k=1}{\sum }}\overset{n}{\underset{j=1}{\sum }}e_{j}^{k}z_{ji}^{kr}{+c}_{i}^{r}$$

where $e_{i}^{r}$ represents the *i*th sector’s embodied energy intensity in province *r*, which shows the total energy consumption for producing its one-unit output. $e_{j}^{k}$ is the *j*th sector’s embodied energy intensity in province *k*. Thereby, $\overset{m}{\underset{k=1}{\sum }}\overset{n}{\underset{j=1}{\sum }}e_{j}^{k}z_{ji}^{kr}$ is the energy use caused by intermediate input of sector *i* in province *r*. $c_{i}^{r}$ stands for the *i*th sector’s direct energy use in province *r*.

Accordingly, *m* × *n* equations similar to Equation (2) are constructed within the whole economy. To simplify the computations, all energy balances are expressed in the form of vectors and matrixes, which is presented as follows:

(3) $$e*\hat{x}=e*Z+c$$

where *e* is the 1 × *mn* row vector of embodied energy intensity. The symbol “^” represents vector diagonalization. *x* is the *mn* × 1 column vector of total output. *Z* is the *mn* × *mn* matrix of intermediate use. *c* is the 1 × *mn* row vector, which represents the direct energy input of each province’s sectors.

Then, the above equation can be further converted as follows:

(4) $$e=c*{\left(\hat{x}-Z\right)}^{-1}$$

In this paper, the TS is the research object. After obtaining the embodied energy intensity, we can analyze the sector’s embodied energy use from the intersector and interprovince perspectives.

(1) Energy use embodied in intersectoral flows

To investigate the energy input–output relations between TS and the other sectors, the intersectoral embodied energy use is measured according to the following equations.

(5) $${sei}_{it}=\overset{m}{\underset{k=1}{\sum }}\overset{m}{\underset{r=1}{\sum }}e_{i}^{r}{*z}_{it}^{rk}$$

where ${sei}_{it}$ represents the EC embodied in inputs from sector *i* to sector *t* (i.e., TS). Then, the energy use embodied in sector’s outputs is also obtained as

(6) $${seo}_{ti}=\overset{m}{\underset{r=1}{\sum }}\overset{m}{\underset{k=1}{\sum }}e_{t}^{r}{*z}_{ti}^{rk}$$

where ${seo}_{ti}$ denotes the EC caused by the demand of sector *i* for the TS.

(2) Energy use embodied in interprovincial transfer

To explore the interprovincial energy transfer of the transport sector, energy embodied in exports and imports is also measured, which will provide an insight into the hidden linkages of the TS among provinces. The formula is presented as follows:

(7) $${IM}_{t}^{r}=\overset{m}{\underset{k\neq r}{\sum }}\overset{n}{\underset{i=1}{\sum }}e_{i}^{k}{*z}_{it}^{kr}$$

where ${IM}_{t}^{r}$ stands for the total energy consumption embodied in the transport sector’s imports in province *r*.

(8) $${EX}_{t}^{r}{=e}_{t}^{r}*(\overset{m}{\underset{k\neq r}{\sum }}\overset{n}{\underset{j=1}{\sum }}z_{tj}^{rk}+\overset{m}{\underset{k\neq r}{\sum }}\overset{p}{\underset{q=1}{\sum }}d_{tq}^{rk})$$

where ${EX}_{t}^{r}$ indicates the total energy consumption embodied in the transport sector’s exports in province *r*.

### 2.2. Data Processing

The latest Chinese MRIO table is available from the research of Mi et al. [46]. It provides the monetary amount of economic interaction for 30 sectors and 30 provinces (The 30 provinces are Beijing (A_1_), Tianjin (A_2_), Hebei (A_3_), Shanxi (A_4_), Inner Mongolia (A_5_), Liaoning (A_6_), Jilin (A_7_), Heilongjiang (A_8_), Shanghai (A_9_), Jiangsu (A_10_), Zhejiang (A_11_), Anhui (A_12_), Fujian (A_13_), Jiangxi (A_14_), Shandong (A_15_), Henan (A_16_), Hubei (A_17_), Hunan (A_18_), Guangdong (A_19_), Guangxi (A_20_), Hainan (A_21_), Chongqing (A_22_), Sichuan (A_23_), Guizhou (A_24_), Yunnan (A_25_), Shaanxi (A_26_), Gansu (A_27_), Qinghai (A_28_), Ningxia (A_29_), and Xinjiang (A_30_)) in 2012. Tibet is not included due to the unavailability of data. Direct energy input data of different sectors in different provinces are obtained and calculated from China Energy Statistical Yearbook 2013 and the statistical yearbook in each province. Meanwhile, energy consumption data are split into seven aspects in each province’s yearbook, i.e., (1) agriculture, forestry, animal husbandry, and fishery; (2) manufacturing; (3) construction; (4) transport, storage, and post; (5) wholesale, retail trade and hotel, and restaurants; (6) other industries; and (7) residential consumption. The category (4) is utilized to represent the corresponding data of the TS, which is consistent with the research of Chung et al. [1].

Although the MRIO table and statistical yearbooks provide detailed data of economy and energy consumption, respectively, sector divisions between the MRIO table and the energy data of some provinces are different. For example, Shanxi, Beijing, Liaoning, and Tianjin provide the EC data of 45 sectors, 57 sectors, 56 sectors, and 37 sectors, respectively, which are not consistent with the MRIO table having 30 sectors. To cope with this inconsistency, two treatment schemes are suggested by Su et al. [47] to decompose and merge sectors to match the MRIO table and the EC data. In the MRIO table, wholesale and retailing sector and hotel and restaurant sector are merged as wholesale, retailing and hotel, restaurants sector; leasing and commercial services sector, scientific research sector, and other services sector are merged as the other services sector. Thus, 27 sectors (The 27 sectors are agriculture (S_1_), coal mining (S_2_), petroleum and gas (S_3_), metal mining (S_4_), nonmetal mining (S_5_), food processing and tobaccos (S_6_), textile (S_7_), clothing, leather, fur, etc. (S_8_), wood processing and furnishing (S_9_), paper making, printing, stationery, etc. (S_10_), petroleum refining, coking, etc. (S_11_), chemical industry (S_12_), nonmetal products (S_13_), metallurgy (S_14_), metal products (S_15_), general and specialist machinery (S_16_), transport equipment (S_17_), electrical equipment (S_18_), electronic equipment (S_19_), instrument and meter (S_20_), other manufacturing (S_21_), electricity and hot water production and supply (S_22_), gas and water production and supply (S_23_), construction (S_24_), transport and storage (S_25_), wholesale, retailing and hotel, restaurants (S_26_),and other services (S_27_)) are determined in this paper. For EC data of each province, we adopt the treatment of Guo et al. [48] to match the revised MRIO table.

## 3. Results

### 3.1. Spatial Distribution of the Embodied Energy Use

**Figure 2:**
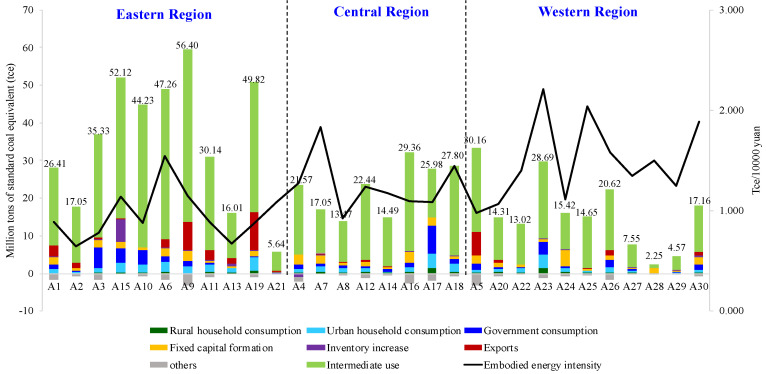
Energy consumption embodied in the transport sector from the perspective of different use.

According to the Equations (1)–(4), the embodied energy intensity is obtained, which can quantify the total EU across the whole supply chain of the TS. Based on it, the TS’s embodied energy consumption caused by different categories of uses can also be calculated, which is shown in Figure 2. Apparently, intermediate use is the major driving factor for the embodied EC in most provinces. It means that the energy embodied in the transport sector is mainly driven by the transfer of products and services among sectors and provinces.

In terms of transport sector’s total energy use, Shanghai (A_9_) consumes 56.4 Mtce in 2012, followed by Shandong (A_15_) with 52.12 Mtce, Guangdong (A_19_) with 49.82 Mtce, Liaoning (A_6_) with 47.26 Mtce, and Jiangsu (A_10_) with 44.23 Mtce. Nevertheless, the driving factors are different for energy increments in these provinces. Except for intermediate use, the contributions of different final uses are various among these provinces. For instance, exports are the main reason driving the EC of the TS (see the red columns in Figure 2) in Shanghai (A_9_) and Guangdong (A_19_). The two regions are the major port provinces in China, where transport activities related to exports are more active. Inventory increase (see the purple columns in Figure 2), government consumption (see the dark blue columns in Figure 2), and urban household consumption (see the light blue columns in Figure 2) are the main factors for EU of the TS in Shandong (A_15_), Jiangsu(A_10_), and Liaoning (A_6_), respectively. Meanwhile, the embodied energy intensity is relatively small in the provinces A_9_, A_19_, A_15_, and A_10_. By contrast, it is higher in A_6_ than those of the other four provinces, which means embodied energy intensity is also an important factor pushing the large amount of transport’s energy consumption in Liaoning. In addition, the ECs of the TS in some provinces are lower while their energy intensities are higher, such as Jilin (A_7_), Sichuan (A_23_), Yunnan (A_25_), Qinghai (A_28_), and Xinjiang (A_30_). A major reason for this phenomenon is that the energy use in these provinces is inefficient due to managerial failure and technical backwardness.

According to the above analysis, it can be seen that the provinces with high ECs maybe have low energy intensities or low ECs with high intensities. In this context, classification of the provinces is necessary for policymakers to make appropriate policies. Traditionally, the provinces in China are grouped into three regions (see Figure 2) based on the geographical positions and the level of economic development. Following this kind of classification, we find that most provinces in the eastern region have high ECs embodied in the TS and relatively low energy intensities, which can be attributed to active economic activities, large population, and advanced technologies. Comparatively, most provinces in the central and western regions have relatively low EU and high energy intensity. This is because that the economy is underdeveloped and transport technology is backward in the two regions.

**Figure 3:**
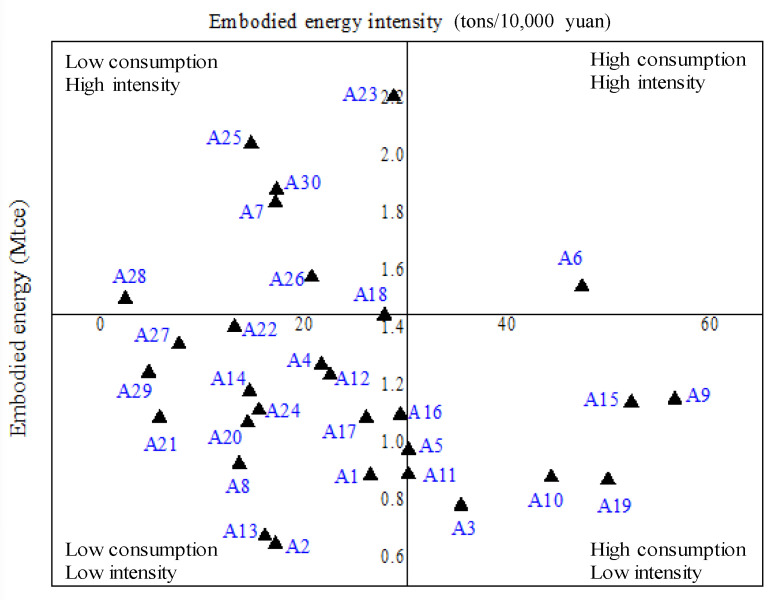
Provinces’ division on the basis of dual criteria.

To further display each province’s EU status of the TS, the provinces are divided into four types based on the embodied EC and the embodied energy intensity, which is shown in Figure 3. Clearly, only Liaoning (A_6_) is located in the quadrant of high consumption–high intensity. Jilin (A_7_), Sichuan (A_23_), Yunnan (A_25_), Shaanxi (A_26_), Qinghai (A_28_), and Xinjiang (A_30_) are in the quadrant of low consumption–high intensity, whose ECs of the TS are mainly caused by higher energy intensity. The TS in Hebei (A_3_), Inner Mongolia (A_5_), Shanghai (A_9_), Jiangsu (A_10_), Zhejiang (A_11_), Shandong (A_15_), and Guangdong (A_19_), which are in the quadrant of high consumption–low intensity, consumes a lot of energy because of the rich economic and social activities.

### 3.2. Energy Use Embodied in Intersectoral Flows and Interprovincial Trade

In this section, the embodied energy consumption of the transport sector is further investigated from the perspectives of transferring with other sectors and among provinces.

#### 3.2.1. Intersectoral Analysis

**Figure 4:**
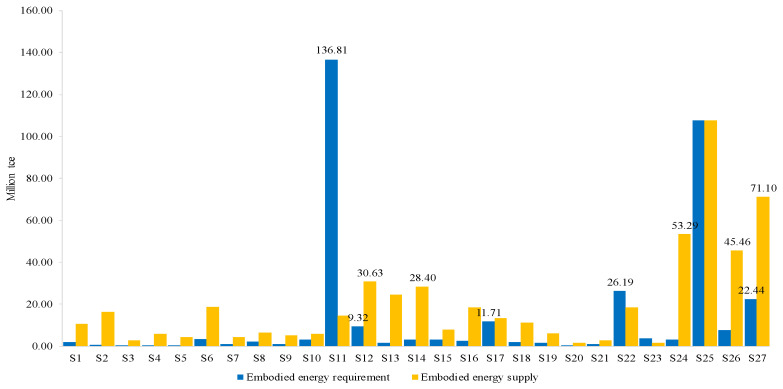
The embodied energy flows of transport sector with other sectors.

First of all, energy flows between transport sector and the other sectors are measured. Figure 4 portrays the transport sector’s energy requirement for the other sectors (i.e., supply side) and its energy supply to the other sectors (i.e., demand side). From the supply side of the TS, except itself (S_25_), most energy is embodied in the inputs from the petroleum refining, coking, etc. sector (S_11_) (136.81 Mtce), followed by the electricity and hot water production and supply sector (S_22_) with 26.19 Mtce, the other services sector (S_27_) with 22.44 Mtce, and the transport equipment sector (S_17_) with 11.71 Mtce. It can be seen that the embodied EU of the TS is mainly offered by the secondary energy production sectors (i.e., S_11_ and S_22_), which is easy to understand. The other services sector (S_27_) is also a major energy contributor for the transport sector. S_27_ includes different kind of services, such as leasing and commercial services, scientific research, banking, real estate trade, education, etc. Among them, some serve the transport sector. For example, leasing and commercial services have subcategories related to transportation, such as the rental of transportation equipment (i.e., cars, railway transportation equipment, boats, and air transportation equipment), and the packaging service for goods. These upstream activities provide services to the transport sector. Energy consumption involved in these activities is the embodied EC of transportation.

From the demand side, most energy is embodied in the inputs from TS to the other services sector (S_27_), the construction sector (S_24_), the wholesale, retailing and hotel, restaurants sector (S_26_), and the chemical sector (S_12_), which contribute 71.10, 53.29, 45.46, and 30.63 Mtce, respectively. The demand of the four sectors for the services of transportation is the main driving force for its embodied energy consumption.

Moreover, it can be seen in Figure 4 that the embodied energy supply is more than the embodied energy requirement except for sectors S_11_ and S_22_. This demonstrates that the transport sector is an embodied energy provider for many sectors. In comparison, the energy sectors (i.e., S_11_ and S_22_) are its energy suppliers. It shows that the transport sector’s energy use has an important impact on the embodied energy use of the other sectors. If the efficiency of the TS’s EU is improved and its energy consumption is reduced, the embodied EU of the other sectors would also be decreased.

#### 3.2.2. Interprovincial Analysis

**Figure 5:**
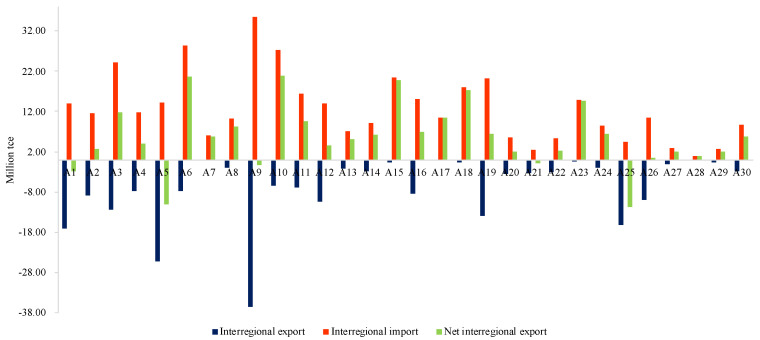
The embodied energy transfer of the transport sector among different provinces.

In this section, the embodied energy transfer of the TS among different provinces is investigated, which is shown in Figure 5. Shanghai (A_9_) is the dominant region. Its energy use embodied in transport sector’s exports to the other regions is the most with 36.59 Mtce, followed by Inner Mongolia (A_5_) with 25.33 Mtce, Beijing (A_1_) with 17.02 Mtce, and Yunnan (A_25_) with 16.20 Mtce. At the same time, Shanghai is also the top region in energy consumption embodied in the transport sector’s imports with 35.4 Mtce, followed by Liaoning (A_6_) with 28.46 Mtce, Jiangsu (A_10_) with 27.21 Mtce, and Hebei (A_3_) with 24.14 Mtce. Additionally, most provinces are the net energy importers except Beijing (A_1_), Inner Mongolia (A_5_), Shanghai (A_9_), Hainan (A_21_), and Yunnan (A_25_), which indicates that the traffic inflows are more than their outflows in many provinces.

**Figure 6:**
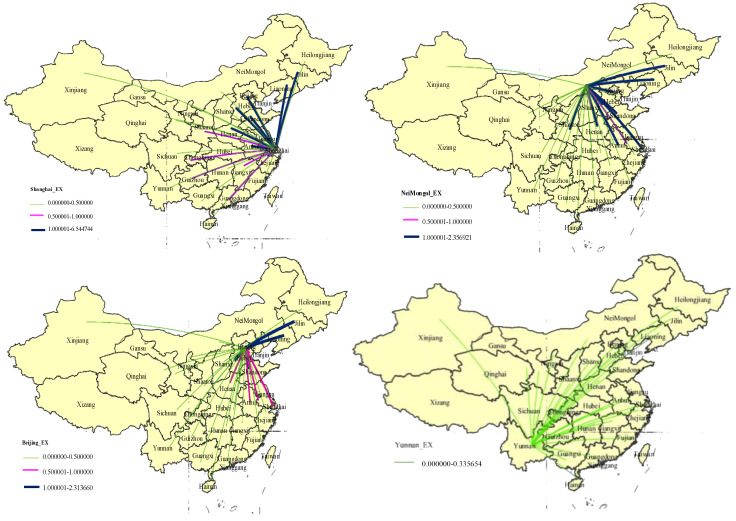
Spatial distribution of energy use embodied in the transport sector exports of the top four provinces in 2012.

To investigate the geographical distributions of energy embodied in the TS’s exports and imports in each province, the top four provinces’ energy transfers are depicted. Figure 6 shows the spatial distribution of energy embodied in the TS’s exports of the top four provinces, which have been identified as Shanghai, Inner Mongolia, Beijing, and Yunnan in the above analysis. For Shanghai, the embodied energy in the TS’s exports is mainly distributed to Jiangsu with 6.54 Mtce, Zhejiang with 4.21 Mtce, Anhui with 3.49 Mtce, and Shandong with 2.20 Mtce, which account for 22.45%, 14.45%, 11.98%, and 7.55% of the total energy output, respectively. Jiangsu, Zhejiang, and Anhui are located in the Yangtze River Delta region. Shanghai, a city also in this area, mainly exports its products and services to other provinces within the area through transportation. For Inner Mongolia, most of its embodied energy of the TS outflows to the Circum-Bohai Sea economic area, i.e., Hebei with 2.36 Mtce, Shandong with 2.01 Mtce, Beijing with 1.81 Mtce, and Liaoning with 1.55 Mtce. They occupy 22.45%, 14.45%, 11.98%, and 7.55% of the total, respectively. For Beijing, most embodied energy of the TS is exported to Shandong with 2.31 Mtce, Hebei with 2.13 Mtce, Liaoning with 1.51 Mtce, and Jilin with 1.19 Mtce. They are also located in the Circum-Bohai Sea economic area. For Yunnan, most energy embodied in its TS’s export is allocated to Guizhou with 0.34 Mtce, Shanghai with 0.25 Mtce, Chongqing with 0.16 Mtce, and Anhui with 0.14 Mtce, which accounts for 36.4% of the total energy outflow of the TS in this province.

**Figure 7:**
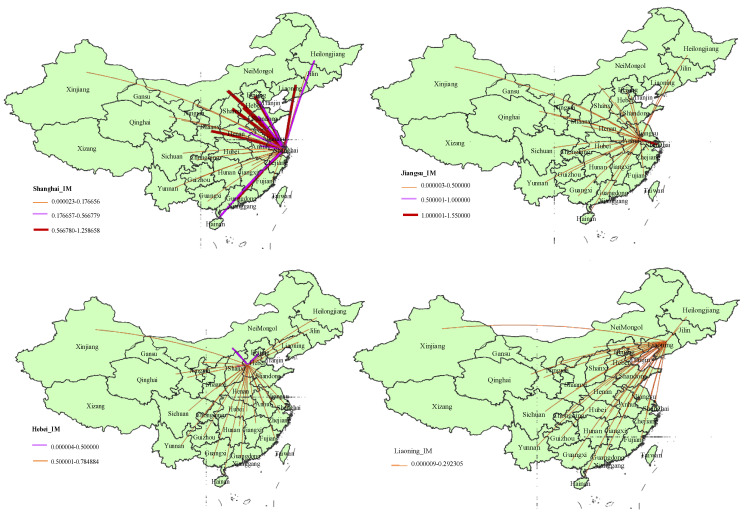
Spatial distribution of energy use embodied in the transport sector imports of the top four provinces in 2012.

The sources of the embodied energy inflows of the TS in the top four provinces are illustrated in Figure 7. For Shanghai, the biggest energy inflows embodied in the TS’s imports are from Inner Mongolia, Zhejiang, Hebei, and Beijing. They provide 38.2% of the total. For Jiangsu province, Shanghai, Inner Mongolia, Anhui, and Hebei are its major energy suppliers, which occupy 60.23% of the total energy embodied in its TS’s import. As for Hebei, the EU embodied in its imports of the TS mainly comes from Beijing with 0.785 Mtce, Inner Mongolia with 0.742 Mtce, Shanxi with 0.681 Mtce, and Shanghai with 0.493 Mtce, which account for 57.15% of the total. In Liaoning, most of the embodied energy of the TS’s imports is mainly from Shanghai with 0.292 Mtce, Hebei with 0.271 Mtce, Inner Mongolia with 0.254 Mtce, and Beijing with 0.204 Mtce, which constitute 45% of the total energy input. Based on the above results, the top four provinces interact more frequently with their neighboring provinces and cities. Both Shanghai and Inner Mongolia are the major source regions for the energy embodied in the imports of the TS of the four provinces.

After analyzing the energy use embodied in exports and imports of each province’s transport sector, there are two main reasons to explain the above spatial distribution.

- The provinces and cities located in the eastern coastal areas have a relatively developed economy, but they are short of resources. To support their abundant economic activities, a lot of petroleum and electricity need to be transferred from the resource-intensive provinces that are located in central and western China. Inner Mongolia, Hebei, and Liaoning are rich in natural resources. In addition, Shanghai is the largest port city in China. It trades goods with other provinces frequently. For many provinces, the energy use embodied in the transport sector’s imports mainly comes from Shanghai because it supplies a lot of products to the other regions through transportation.
- Based on the spatial distributions of the embodied energy transfers of the TS, we find a geographically close phenomenon between a province and its frequently exchanging regions. It is easy to understand that a close geography can facilitate the transportation of products and services. Thus, provinces are more likely to interact with the neighbors.

## 4. Discussions and Policy Implications

### 4.1. Discussions

- Sectoral perspective

**Figure 8:**
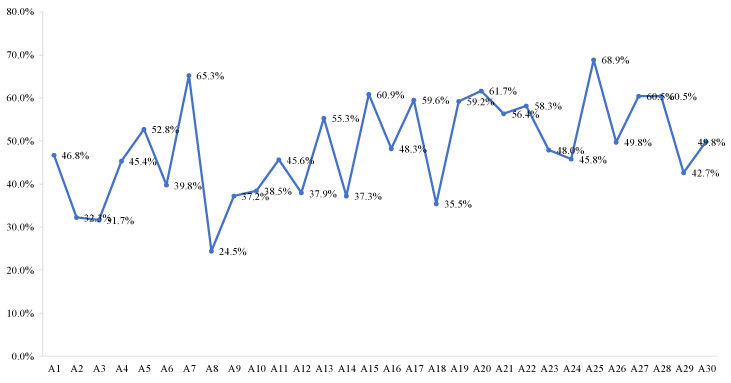
Proportion of direct energy use in the embodied energy use of transportation sector.

The embodied energy consumption of the TS has been measured and analyzed in the above section. To further explore the energy use, it is essential to take the basic characteristics of transport activities into account. Figure 8 shows the proportion of direct energy use in the transport sector’s total energy consumption. The ratios change from 24.5% in Heilongjiang to 68.9% in Yunnan, and most of them are in the interval [40%, 60%]. As we all known, transportation is a tool for the transfer of various products, people, and services. From the perspective of the whole supply chain, the direct EU occurs during transportation; while the indirect EU is caused by its supply and demand. Specifically, in the term of its demand, the direct EU is relevant to the production of transport equipment, petroleum, electricity, and other products and services carried on vehicles. According to the results of Section 3.2.1, the petroleum refining, coking, etc. sector (S_11_), the electricity and hot water production and supply sector (S_22_), the other services sector (S_27_), and the transport equipment sector (S_17_) are the top four embodied energy suppliers of the transport sector. They are located in the sector’s upstream supply chain producing fuel and electricity, providing related leasing and legal services, and manufacturing transport equipment. Therefore, optimizing the structure of energy production (such as using clean energy) and upgrading intersectoral economic relationship are feasible methods to improve the EU embodied in the TS. Meanwhile, in the term of the supply to the TS, a lot of energy is embodied in the outflows of the transport sector to the other services sector (S_27_), the construction sector (S_24_), the wholesale, retailing and hotel, restaurants sector (S_26_), and the chemical sector (S_12_), which is more than the inflows from these sectors. Hence, decreasing the energy intensity of the TS can indirectly reduce the embodied energy consumption of the four sectors, and then, the whole supply chain.

- Provincial perspective

In Section 3.1, the provincial heterogeneity of the embodied energy use of transport sector was analyzed. The energy intensity of the TS was high in a lot of provinces, such as Liaoning, Jilin, Sichuan, and Yunnan, which can be reduced by improving their energy efficiency, such as adopting advanced energy technologies and vehicles. The TS has relatively low energy intensity and massive EC in some provinces, such as Beijing, Shanghai, Jiangsu, and Guangdong. This can be attributed to the high volumes of economic and transport activities in these regions [49]. However, limiting transport activities is infeasible in the background of the rapid growth of economy, urbanization, and construction of transport infrastructure in China. Thus, there are some other ways to constrain the fast energy consumption of transportation, for instance, encouraging public transport, increasing fuel taxes, and promoting more green transportation modes.

Further, EU embodied in the interprovincial trade of the TS was analyzed, which provides a deeper insight of the current status of the TS’s EU. Based on the analysis in Section 3.2.2, there are two phenomena in the interprovincial energy flows of the TS: one is that they are transferred from the energy-intensive regions to the energy shortage areas; the other one is that they interflow among the provinces with close geographies. For example, Inner Mongolia has been recognized as a major energy provider for many regions. From the view of producer, the transport sector in this province should be constrained through the implementation of strict energy policies. By contrast, the exploration of the TS’s energy transfer in this paper gives decision-makers a consumer-based perspective to make policies, which would require the provinces with net transportation demand to take more responsibilities in declining the EC of the TS.

### 4.2. Policy Implications

According to the results, intermediate use is a major factor for the TS’s EU, which is related to massive economic activities in China. From the perspective of final uses, consumptions of the household and government are also major contributors to the TS’s embodied energy use. It is consistent with the increase of resident income, urbanization, and transport infrastructure construction in China. In the future, the economic development and urbanization in China will continue to grow. Thus, it is significant to conduct reasonable and fair energy policies for the transport sector to control the increase of energy consumption, where sectoral and regional transfers are both considered. In the following, policies are suggested from the sectoral and the regional perspectives, respectively.

- Sectoral policy

Sectoral policies improving the TS’s embodied EU could be proposed from the perspective of its supply and demand. In terms of the TS’s supply side, the basic strategies include optimizing energy structure, improving energy efficiency of the TS’s suppliers, and making the production process low carbon. Results have revealed that petroleum produced in S_11_ is the dominant embodied energy for the TS in China. However, petroleum is a non-renewable energy, and its combustion can emit a lot of pollutions. Although many cities in China have used and encouraged residents to choose the electric vehicles to replace fuel ones, nearly 78.1% of electricity in China is generated from coal-based thermal power in 2012. In this respect, the energy structure should further be optimized through increasing the percentage of renewable energy, such as wind energy, photovoltaic, and nuclear energy, to produce electricity. Moreover, governments could support manufacturing companies of transportation equipment to improve their energy efficiency, reduce energy intensity, and produce renewable energy-based automobiles by way of subsidies and R&D investment.

In terms of the TS’s demand side, it is unrealistic to decrease other sectors’ demand for the TS in the context of sustained economic development. Therefore, reducing the embodied energy intensity of the TS itself is a feasible way. Transportation is at the upstream for many sectors. Improving its efficiency of EU and guiding the green development can affect other sectors to be greener.

- Regional policy

Based on the provinces’ division in Figure 3, several strategies are summarized and proposed for different regions to improve the EC of the TS. They are divided into two types, i.e., strategies for high embodied energy intensity and strategies for high embodied EC. Provinces can refer to either one or two based on the EC status of their TS.

For provinces with a high embodied energy intensity of the TS, three strategies are provided and presented as follows: First, most of them use traditional vehicles that consume energy inefficiently. In comparison, the provinces with relatively low energy intensity, e.g., Guangdong, have promoted energy-efficient vehicles with advanced technologies, such as natural gas vehicles and electric vehicles. Hence, the provinces can adopt advanced vehicles from the developed ones through technology transfer. Second, provinces have more economic and social exchanges with their neighbors. The EC embodied in these activities could be reduced through optimizing the transport modes, because different transport modes have different EC levels. For example, the EC of road transport is higher than that of railway. Although high-speed railway is booming in China, the railway capacity is still inadequate especially in the underdeveloped regions. In this respect, the highway is often chosen by people in these regions for long-distance delivery of goods or passengers. Thus, increasing the percentage of railway transport can effectively improve the current energy intensity of the TS. Third, optimizing the production structure of the whole economy is an effective way to reduce the embodied energy intensity. Governments could promulgate policies, such as subsidies and taxes, to encourage industries to utilize energy-efficient and resource-efficient materials and technologies and to abandon inefficient production. As a result, the embodied energy intensity of the TS can be decreased due to the declining of EC embodied in the upstream of the TS.

For the provinces with high EC, economic development is the main cause, except for high energy intensity. However, economic development is inevitable in China. Thus, four practices are provided without affecting the economy growth. First, for the developed provinces, lots of transport activities from resource-rich area are required due to the high energy demand. These provinces can use clean energy, such as wind energy, solar energy, nuclear energy, and water energy to meet local energy demand. Therefore, the energy embodied in transportation inflow from other regions can be decreased. Second, the local government should further develop public transport systems, e.g., high-speed rail, buses, light rail, and subway. Meanwhile, private fuel cars should be limited through the promotion of new energy vehicles (NEVs) and the implementation of some economic measures, such as fuel taxes and emission charges. Third, governments can improve the public awareness of environmental protection and energy saving through various means, such as advertisement on TV, newspapers, and the internet. Fourth, the energy allocation efficiencies of the TS can be improved by governments through the reform of energy price mechanism and by improving the management efficiency of NEVs. In China, energy price in the transport sector is determined by the government, which would cause price distortions and inefficiency of resource allocation [50,51]. Moreover, in many developed Chinese cities, NEVs development is affected by inefficient management, for example, local protectionism, insufficient market momentum, multiple managements, inefficient subsidy policies, and low utilization of charging facilities due to layout and structural issues [52]. Solving these issues can further improve the energy efficiency of the TS and decrease its EC.

## 5. Conclusions

With the continuous development of the economy, the transport sector has been one of the major energy consumption departments in China. It is of great significance to investigate its current status of energy consumption and reduce pollutant emissions. In this study, a MRIO model is utilized to estimate the total energy consumption of China’s transportation sector, which provides spatial and sectoral perspectives for policymakers to have a deeper insight of its energy use. The main conclusions are listed as follows:

- Intermediate use contributes the most to the energy consumption of the transport sector in most provinces, which indicates that the transport sector mainly serves in the delivery of products and services among sectors and provinces. The contributions of final uses are various in different provinces, where consumptions of households and government are the main factors in all provinces.
- Regional heterogeneity is one of the features in the TS’s EU in China. In most eastern provinces, the transport sector has high embodied EC and relatively low embodied energy intensity, while most provinces in central and western regions have relatively low embodied EU and high embodied energy intensity. Further, the provinces are divided into four types based on the embodied EC and the embodied energy intensity. Such division would help central and provincial governments to implement measures specific to each province.
- From the supply side of the TS, most EU was embodied in supplies from the petroleum refining, coking, etc. sector (S_11_), the electricity and hot water production and supply sector (S_22_), the other services sector (S_27_), and the transport equipment sector (S_17_). From its demand side, the TS offers most of the embodied energy to the other services sector (S_27_), the construction sector (S_24_), the wholesale, retailing and hotel, restaurants sector (S_26_), and the chemical sector (S_12_). Additionally, the TS is a net embodied energy supplier to many other sectors, which demonstrates that the embodied EU of other sectors would be decreased if the energy consumption of transportation can be reduced.
- From the perspective of interprovincial trade, energy embodied in the TS flows from resource-intensive provinces located in central and western China to resource-deficient provinces in eastern China. Being the largest port city in China, Shanghai exports the most embodied energy of the TS. In addition, a close geographical connection exists in the interprovincial energy transfer embodied in the TS.
- Finally, policy suggestions are provided to improve the status of the transport sector’s energy consumption, such as adopting advanced technologies and vehicles, optimizing transport modes and energy structure, and increasing public awareness of energy conversion, instead of reducing transport activities.

In conclusion, there are some limitations that need to be improved in the future, which are listed as follows: (1) The transport sector is analyzed as a whole in this paper and different types of transportation, e.g., roads, railways, water transport, and air transport, are not discussed. However, the different types of transport have different characteristics, and these heterogeneities would be considered in further studies. (2) Only a MRIO table from 2012 is used in this paper, in the future, more tables issued in the previous years would be utilized to explore the embodied energy consumption trend of the transportation sector in China.

## Figures and Tables

**Table 1:** The revised format of the MRIO table.

	Output	Intermediate Use										Final Use						Total Output
Input		Province 1 (A_1_)			Province 2 (A_2_)			…	Province m (A_m_)			Domestic Final Use				Foreign Demand	Others	
		S_1_	…	S_n_	S_1_	…	S_n_		S_1_	…	S_n_	Province 1 (A_1_)	Province 2 (A_2_)	…	Province m (A_m_)	Exports		
Province 1 (A_1_)	S_1_	$z_{ij}^{rs}$										$d_{iq}^{rs}$				$e_{i}^{r}$	$o_{i}^{r}$	$x_{i}^{r}$
	S_2_																	
	…																	
	S_n_																	
Province 2 (A_2_)	S_1_																	
	S_2_																	
	…																	
	S_n_																	
…																		
Province m (A_m_)	S_1_																	
	S_2_																	
	…																	
	S_n_																	
Direct energy input		$c_{j}^{s}$																

## Data Availability

The data of Chinese MRIO table used in this study are openly available at [https://doi.org/10.1038/s41467-017-01820-w, accessed on 30 May 2021], reference number [46]. The direct energy input data are from publicly available datasets, which can be found here: [https://data.cnki.net/yearbook/Single/N2021050066, accessed on 30 May 2021].
